# Editorial: Radiomics and radiogenomics in genitourinary oncology: artificial intelligence and deep learning applications

**DOI:** 10.3389/fradi.2023.1325594

**Published:** 2023-12-18

**Authors:** Alessandro Stefano, Elena Bertelli, Albert Comelli, Marco Gatti, Arnaldo Stanzione

**Affiliations:** ^1^ Institute of Molecular Bioimaging and Physiology, National Research Council (IBFM-CNR), Cefalù, Italy; ^2^ Department of Radiology, Careggi University Hospital, Florence, Italy; ^3^ Ri.MED Foundation, Palermo, Italy; ^4^ Department of Surgical Sciences, University of Turin, Turin, Italy; ^5^ Department of Advanced Biomedical Sciences, University of Naples “Federico II”, Naples, Italy

**Keywords:** radiomics, radiogenomics, genitourinary oncology, artificial intelligence, computer-aided design system, testicular imaging, renal cell carcinoma, prostate cancer

**Editorial on the Research Topic** Radiomics and radiogenomics in genitourinary oncology: artificial intelligence and deep learning applications

This Research Topic aimed to promote innovative approaches to radiomics and radiogenomics analyses in genitourinary oncology, utilizing artificial intelligence (AI). While quantitative methods have been used in oncologic imaging, the multi-step process known as radiomics has transformed medical images into a valuable source of data, with the potential to advance precision medicine (1). Radiomics operates on the premise that image heterogeneity at the voxel level can mirror the biological heterogeneity of tumors at a deeper level. In this way, an extensive array of quantitative parameters can be extracted from all commonly used clinical imaging modalities, offering numerous possibilities for discovering new biomarkers. These high-dimensional datasets can be efficiently and effectively managed using AI. Indeed, machine learning (ML) and deep learning (DL) can be used to build radiomics-powered predictive models and decision support tools (2).

Radiogenomics combines radiomics with genomics by exploring potential links between tumor gene expression and imaging features (3).

Unfortunately, the translation of radiomics and radiogenomics into clinical practice is proving rather difficult to achieve (4). Radiomics pipelines need to be sufficiently robust, and generalizability is a major concern (5). Indeed, high dimensionality and the risk of overfitting undermine the findings of many preliminary investigations. Issues related to each step of radiomics workflow, from segmentation (e.g., feature stability) (6) to model validation (e.g., the need for external datasets) must be addressed (7). Nevertheless, the promises of radiomics in the field of genitourinary oncology are definitively worthy of being explored. Radiomics models might increase the diagnostic accuracy of imaging modalities in the detection of tumors, aid radiologists in the characterization of imaging findings, and even predict treatment response or patient prognosis.

The topics of interest of this Research Topic included:

• Development of radiomics-powered DL tools, e.g., computer-aided design (CAD) systems
• Reproducibility of radiomics features in genitourinary tumors
• Development of diagnostic/prognostic models with state-of-the-art radiomics pipelines and AI techniques
• Validation of previously published radiomics signatures
• Evaluation of the clinical applicability of radiomics models
• Investigating correlations between radiomics features and genitourinary tumors gene expression
• AI applications to radiomics in genitourinary oncology, specifically original articles, and reviews.

In response to the call for papers, a total of eight submissions were accepted for publication in this Research Topic, comprising two reviews, respectively on testicular imaging (Fanni et al.) and renal cell carcinoma (RCC) (Wang et al.), as well as six original articles. Of the latter, two investigated bladder cancer (BCa) applications (Chen et al., Deng et al.), while four were focused on prostate cancer (PCa) (Huang et al., Lu et al., Li C et al., Li L et al.).

Concerning the reviews, the first one by Fanni et al. aims to assess the current state of radiomics in testicular imaging by evaluating the quality of radiomics workflows using the Radiomics Quality Score (RQS) and the Quality Assessment of Diagnostic Accuracy Studies-2 (QUADAS-2). A systematic literature search was then conducted to identify potentially relevant studies concerning the utilization of radiomics in testicular imaging, resulting in the inclusion of six papers. The mean RQS was 11.33 ± 3.88, equating to a percentage of 31.48% ± 10.78%. Regarding the QUADAS-2 criteria, no significant biases were detected in the included papers in the domains of patient selection, index test, reference standard criteria, and flow-and-timing. In conclusion, despite the emergence of promising studies, the field of radiomics in testicular imaging is still in its infancy, constrained by methodological limitations.

The second study by Wang et al. presents a comprehensive review of the latest advancements, challenges, and prospects in the application of DL for diagnosing RCC. Notably, it is the first review of DL role in RCC diagnosis. The review underscores the immense potential of DL in the realm of RCC diagnosis, anticipating further research outcomes that will ultimately benefit RCC patients. Given that medical imaging is pivotal for early RCC detection, as well as for monitoring and assessing RCC throughout treatment, the digitalization of commonly used technologies like contrast-enhanced computed tomography (CECT), ultrasound, and magnetic resonance imaging (MRI) has opened the door for DL applications allowing clinicians to achieve more accurate and faster diagnoses of renal tumors.

Concerning the original articles, the first one by Chen et al. addresses the challenges associated with the preoperative diagnosis of BCa and its assessment of muscular invasion using Computerized Tomography (CT) images. A DL radiomics signature was developed for this purpose, involving a retrospective analysis of 173 patients, with 43 having pathologically confirmed muscle-invasive Bca and 130 with non-muscle-invasive Bca. The patients were divided into training and test cohorts, consisting of 129 and 44 patients, respectively. The study employed statistical techniques such as the Pearson correlation coefficient and the least absolute shrinkage and selection operator (LASSO) to reduce feature redundancy. Principal Component Analysis was used to reduce the dimensionality of DL features. Among the six ML classifiers used to predict the muscle invasion status of Bca, the results demonstrated that the Multilayer Perceptron model performed the best. In conclusion, the study highlights the effectiveness of a deep radiomics model constructed using CT images in accurately predicting the muscle invasion status of Bca, which has significant implications for preoperative assessment and treatment planning.

The second study by Deng et al. aims to develop a radiomics model that integrates multiple clinical features to predict the pathological grade of BCa before surgery using non-enhanced computed tomography (NE-CT) images in a retrospective analysis of 105 patients, comprising 44 low-grade and 61 high-grade BCa cases. Patients were randomly split into training (*n* = 73) and validation (*n* = 32) groups in a 7:3 ratio. A total of 15 most correlated with BCa grade were identified using the LASSO algorithm. Six predictive models for BCa pathological grade were implemented, including support vector machine (SVM), k-nearest neighbor (KNN), gradient boosting decision tree (GBDT), logistic regression (LR), random forest (RF), and extreme gradient boosting (XGBOOST). Additionally, a model that combined radiomics and clinical factors was developed. The SVM model achieved the highest area under the receiver operating characteristic curve (AUC) of 0.842. A nomogram combining radiomics and selected clinical variables accurately predicted BCa pathological grade preoperatively, with an AUC of 0.919 for the training group and 0.854 for the validation group. In this way, a non-invasive and accurate preoperative assessment method for BCa patients has been proposed.

The third original article by Huang et al. discusses the potential of ML techniques in improving the precision, consistency, and efficiency of PCa histopathology diagnosis, which traditionally relies on analysis of biopsy tissues. The study introduces a novel classification fusion network model that combines eight advanced image features, including both hand-crafted and DL features. When tested on 1,100 prostate pathology images using various backbones (ResNet-18/50, VGG-11/16, and DenseNet-121/201), the fusion model with the ResNet-18 backbone demonstrated the highest performance in terms of accuracy (95.54%), specificity (93.64%), sensitivity (97.27%), and AUC (98.34%). Notably, individual assessment criteria for each of the distinct features had values lower than 90%, indicating that the proposed model effectively combines different features in an effective manner. Furthermore, the study used a Grad-CAM++ heatmap to visualize the differences between the proposed fusion network and ResNet-18 in terms of identifying cancerous cells. The heatmap revealed that the proposed model outperformed ResNet-18. In conclusion, the classification fusion network, which amalgamates hand-crafted and deep-learning features, holds promise for computer-aided diagnosis based on Pca pathology images.

In the fourth work (Lu et al.), build a radiomics model based on biparametric prostate MRI to detect PCa in patients with PSA serum levels ranging from 4 to 10 ng/ml. The authors of this single-center retrospective study trained and internally tested seven different machine learning algorithms on their cohort (*n* = 136) to finally obtain a radiomics signature. The results are promising, and the study has the merit of having explored clinical parameters (i.e., transition zone PSA density) to build a holistic model. Indeed, the best performance was achieved by the radiomics nomogram which reached an AUC of 0.872. Independent validation of the nomogram is warranted in order to assess its generalizability and it deserves to be reminded that experts in the fields are currently challenging the role of nomograms in radiomics applications (8).

With a different approach to a similar diagnostic challenge (i.e., diagnosis of PCa), rather than integrating clinical data in their model as in the previous study, Li C et al. combined hand-crafted radiomics and DL to maximize the performance in terms of cancer detection. While it could be argued that an unavoidable degree of redundancy would be present in such strategy, the combined model resulted as the most informative suggesting that there might be an advantage in analyzing medical images with different techniques simultaneously. It is also interesting to note how T2-weighted and diffusion weighted imaging (DWI) were once again selected, which is in line with their role as dominant sequences in current imaging guidelines although the recommended imaging protocol still includes a dynamic contrast enhanced sequence; it could be speculated that such choices reflect the trend of growing interest toward biparametric prostate MRI (9). Overall, considering the relatively small sample size (study population of 236 patients) and the lack of external validation, caution is warranted in the interpretation of these noteworthy results.

Finally, in the sixth original work Li L et al. evaluate the performance of multiple classifiers in differentiating patients with PCa from patients with benign lesions. The population was composed by 290 lesions in 238 patients and the Authors classified their results in a patient-based classification and in a lesion-based one. They constructed 4 radiomics models for each group, 3 models were based on T2WI, on apparent diffusion coefficient (ADC) maps and on DWI sequence respectively and the last one was a hybrid model based on some features extracted from all the previously mentioned sequences. ADC and DWI models performed better than T2WI models, whether on patient-based or lesion-based datasets. Furthermore, on ADC and DWI the classification performance on a single-lesion-based classifier is higher than that of a patient-based classifier. Among the 6 classifiers tested, the RF performed better. These results confirmed data reported in Literature (10, 11).

It is interesting to underline, as previously notes also for the work by Li C et al. that the best performance is achieved with the models based on DWI, that is the dominant sequence for the peripheral prostate zone. The main limitations of the study are the lack of an external validation [but unfortunately, it's a common limitation, as previously reported in (Li C et al.)] and that the Authors didn't divide the lesions in peripheral and transitional ones, but they explained this choice in the Discussion.

We hope that the reader will find in this Research Topic a useful reference for the state of the art in the emerging field of radiomics applied to genitourinary oncology.
